# Prevalence and determinants of anxiety in patients with epilepsy during COVID-19 pandemic

**DOI:** 10.1186/s41983-022-00513-6

**Published:** 2022-06-23

**Authors:** Mohammad Gamal Sehlo, Wafaa Samir Mohamed, Usama Mahmoud Youssef, Shrouk Esam Lotfi, Ghada Mohamed Salah El-deen

**Affiliations:** 1 Psychiatry Department, Faculty of Medicine, Zagazig University, P.O. Box 44519, Zagazig, Egypt; 2 Neurology Department, Faculty of Medicine, Zagazig University, Zagazig, Egypt; 3 Neuropsychiatry Resident at Abbaseya Hospital for Mental Illness, Cairo, Egypt

**Keywords:** COVID-19, Epilepsy, Anxiety

## Abstract

**Background:**

Epilepsy is one of the most frequent and serious brain disorders. The nature of the disorder and the unpredictability of seizures usually puts patients in a state of apprehension and anticipation, which creates a continuous condition of anxiety. COVID-19 pandemic has created a state of generalized anxiety all over the world. It is expected that patients with epilepsy (PWE) will suffer from more anxiety during the pandemic. This cross-sectional study was applied on 290 PWE. Data were collected by personal interview with each patient using GAD-7 scale for diagnosing anxiety and assessing its severity. We aimed to assess the prevalence of anxiety and to assess its risk factors in PWE during COVID-19 pandemic.

**Results:**

We found that 52.4% of PWE suffered from anxiety. Not working, low financial status, fear of infection and death by COVID-19, fear of job loss, had job changes during pandemic, increased seizures rate during pandemic, increased ER visits, and lack of drug adherence during the pandemic, are significantly associated with increased risk of anxiety.

**Conclusions:**

COVID-19 pandemic has a serious effect on the psychological and the physical wellbeing of PWE. There was an increased rate of anxiety during COVID-19 pandemic in PWE with its subsequent burden on those patients. So, these patients are in a high need of care and support during the pandemic.

## Background

According to International League Against Epilepsy (ILAE), epilepsy is defined as two unprovoked seizures more than 24 h apart. Epilepsy is now better classified as a disease rather than a disorder because the term disease implies a longer-term disruption of normal function [1].

Psychiatric illness is over-represented in epilepsy as compared with other chronic medical illnesses [2].

One out of every three epilepsy patients will develop a psychiatric disorder at some point in their lives, with mood and anxiety disorders being the most common [3].

Reduced quality of life, greater risk of suicide, poorer response to anti-epileptic drugs (AEDs), increased seizure activity and severity are all associated with anxiety comorbidity in epilepsy [4].

The pandemic of COVID-19 resulted in a significant higher level of anxiety. People began to be concerned about the future because of the virus’s predicted course and spread. The prospect of becoming infected, as well as the media coverage, creates a lot of tension and anxiety. Closures, loneliness as a result of home quarantine, and financial reasons all contribute to distress [5].

Patients with chronic conditions like epilepsy are expected to have more anxiety during the pandemic.

The prevalence of anxiety symptoms during the pandemic across 11 studies ranged from 6.33 to 50.9% [6].

Anxiety in PWE during the pandemic can be attributed to multiple risk factors. Van Hees and colleagues, (2020) found that anxiety in PWE is associated with financial problems during the pandemic [7], Rudenstine and colleagues., (2021) found that COVID-19-related experiences, such as unemployment, the death of a family member or close friend, lack of social support, difficulty paying monthly rent, and income insecurity, increase the scores of anxiety in PWE [8]. Salari and colleagues, (2020) found that the anxiety levels in PWE during the pandemic were higher if one of the relatives had been infected with COVID-19, and in patients with disturbed sleep pattern [9]. Liu and colleagues, (2020) found that increased seizure frequency during the pandemic is associated with increased anxiety [10].

Our study focuses on investigating the prevalence and risk factors of anxiety in patients with epilepsy (PWE) during COVID-19 pandemic.

To our knowledge, this is the first study that had been conducted to assess anxiety among PWE during COVID-19 pandemic in Egypt.

## Methods

A convenience sample of 290 patients diagnosed with epilepsy according to International League against Epilepsy (ILAE) classification 2017, were included in this cross-sectional study. The patients were recruited from the outpatient clinic and the inpatient ward of the Neurology Department, in a University Hospital in Egypt, between August 2020 and September 2021.

Both male and female patients with an age range from 19 to 60 years were included in the study. A written consent was obtained from all the participants to approve their participation in the study.

Exclusion criteria were patients with pseudo-seizures, substance abuse, intellectual disability, and patients with chronic major medical disorders other than epilepsy, patients with previous or current affection with COVID-19. The following measures were applied: 1—Sociodemographic and clinical data form: that is composed of questions related to personal and clinical characteristics of the patients and questions related to COVID-19 pandemic, including age and gender, marital status, employment status, number of children, educational degree, financial status, where and with whom he lives, family history of epilepsy and psychiatric illness, epilepsy-related data; type of seizures, response to AEDs (respondent or resistant)**,** age of onset, time of seizure occurrence, number of drugs, rate of seizures before and during the pandemic, number of previous ER visit by a seizure, number of ER visits by a seizure during the pandemic, fear of having uncontrolled seizure during the pandemic, drug adherence during the pandemic, routine follow-up during the pandemic. COVID-19-related data: close people infection or death, following news about the pandemic, sleep disturbance during the pandemic, family support during the pandemic, job changes during the pandemic, financial changes during the pandemic, fear of job loss during the pandemic, fear of infection or death by COVID-19, fear of close one’s infection or death by COVID-19, sense of the end of the world. 2—GAD-7 (Generalized Anxiety Disorder 7): the 7-item Generalized Anxiety Disorder scale (GAD-7) [11] was used to assess the anxiety symptoms. The GAD-7 measures the severity of anxiety symptoms experienced during the past 2 weeks on a scale from 0 (not at all) to 3 (nearly every day), with a scale range of 0–21. Mild, moderate and severe anxiety is indicated by scores of 5, 10, and 15, respectively. The psychometric properties of the scale are well-established and internal reliability is high (alpha = 0.91). Although originally developed for GAD, the GAD-7 also proved to have good sensitivity and specificity as a screener for panic, social anxiety, and post-traumatic stress disorder [12]. A validated Arabic version of the scale was used in the study [13].

### Statistical analysis

The data analysis and sample size calculation (with 80% power) were performed using the Statistical Package for Social Sciences (SPSS version 25) released in 2017, created by IBM, Armonk, New York, USA [14]. The categorical data were presented in the form of number and percentage. Continuous data were expressed as mean ± SD (standard deviation) and median with the interquartile range (IQR). Chi-square was used as a test of significance of the differences among groups. Binary logistic regression analysis was used to assess the predictors of depression. A *P* value < 0.05 was considered to indicate statistical significance.

## Results

Our results showed that the mean age of the studied group was 33.69 years. More than half of them were male (52.4%). About 50% of them live in urban area, 55.9% live with their spouse and siblings. 56.2% of them were married. 54.1% had secondary education. 40.3% of them were working. Low financial status was found among 39% of them, while financial status was satisfying among 54.1% of them. Finally, 37.6% of them had no children and 39.6% had 1–2 children (Table 1).

**Table 1:** Demographic characteristics of the studied group

Variable		(*n* = 290)
Age: (years)	Mean ± SD	33.69± 9.14
	Range	18–60

Variable		*n*	%
Sex	Female	138	47.6
	Male	152	52.4
Residence	Urban	145	50
	Rural	145	50
Live with	Alone	12	4.1
	Spouse and siblings	162	55.9
	Parents	102	35.2
	Brothers and sisters	10	3.4
	Sibling only	4	1.4
Marital status	Single	96	33.1
	Married	163	56.2
	Widow	6	2.1
	Divorced	25	8.6
Education	Illiterate	44	15.2
	Secondary	157	54.1
	University	80	27.6
	Post-graduate	9	3.1
Occupation	Not working	173	59.7
	Working	117	40.3
Financial status	Low	113	39
	Satisfying	157	54.1
	High	20	6.9
Number of children	n	109	37.6
	1–2	115	39.6
	> 2	66	22.8

*SD* standard deviation

Table 2 shows that the median age of onset of epilepsy among the studied group was 17 years while duration of epilepsy was 14 years. About 21.4% of them had positive family history of epilepsy, 2.8% had positive family history of psychiatric disease and 8.3% had positive past history of psychiatric disease. The most frequent type of seizures found among the studied group was generalized (43.4%) also 69.3% of the cases were respondent to the treatment. Almost 89% of the studied group received more than 1 AEDs. Finally, 83.3% of the cases had seizures at any time.

**Table 2:** Clinical data of epilepsy

Variable		(*n* = 290)
Age of onset (years)	Median (IQR)	17 (10–24)
Duration (years)	Median (IQR)	14 (7–23)

Variable		*n*	%
Family history of epilepsy	Negative	228	78.6
	Positive	62	21.4
Family history of psychiatric disease	Negative	282	97.2
	Positive	8	2.8
Past history of psychiatric disease	Negative	266	91.7
	Positive	24	8.3
Type of seizures	Focal	96	33.2
	Generalized	126	43.4
	Focal with secondary generalization	68	23.4
Response	Respondent	201	69.3
	Refractory	89	30.7
Number of AEDs	1	33	11.4
	> 1	257	88.6
Time of seizures	Any time	243	83.8
	Day	26	9
	Night	21	7.2

*IQR* interquartile range

Our results showed that 26.2% had moderate anxiety and 19.3% had severe anxiety according to GAD 7 score (Table 3).

**Table 3:** Prevalence of anxiety

GAD 7 score	Mean ± SD	8.48 ± 5.81
	Median (IQR)	7.5 (3–14)
	Range	0–19
	No *n* (%)	138 (47.6%)
	Mild *n* (%)	20 (6.9%)
	Moderate *n* (%)	76 (26.2%)
	Severe *n* (%)	56 (19.3%)

*SD* standard deviation, *IQR* interquartile range

There was a statistically significant increase in frequency of severe anxiety among patients fearing COVID-19 infection and death, those who fear from job loss, those who already had job changes by pandemic including job loss, patients who having sleep disturbances during the pandemic and those who had a decrease in family support, and those who were a continuous follower of pandemic news (Table 4).

**Table 4:** The relationship between anxiety and COVID-19

Variable		*n*	None		Mild to moderate		Sever		*χ*^2^	*P*
			(*n* = 138)		(*n* = 96)		(*n* = 56)			
			*n*	%	*n*	%	*n*	%		
Fear of infection by COVID 19	No	136	90	66.2	45	33.1	1	0.7	64.36	0.001*
	Yes	154	48	31.2	51	33.1	55	35.7		
Fear of death by COVID 19	No	125	69	55.2	42	33.6	14	11.2	10.18	0.006*
	Yes	165	69	41.8	54	32.7	42	25.5		
Close people infection	No	135	71	52.6	39	28.9	25	18.5	2.77	0.25
	Yes	155	67	43.2	57	36.8	31	20		NS
Close people death	No	272	124	45.6	93	34.2	55	20.2	5.59	0.08
	Yes	18	14	77.8	3	16.7	1	5.6		NS
Fear of job loss in pandemic	No	203	106	52.2	82	40.4	15	7.4	62.96	< 0.001**
	Yes	87	33	37.9	14	16.1	41	47.1		
Job changes by pandemic	No	205	109	53.1	82	40	14	6.8	71.05	< 0.001**
	Yes	85	29	34.1	14	16.5	42	49.4		
Financial changes in pandemic	No	62	30	48.4	22	35.5	10	16.1	0.56	0.76
	Yes	228	108	47.4	74	32.5	46	20.2		NS
Sleep disturbance during pandemic	No	213	117	54.9	74	34.7	22	10.4	17.62	< 0.001**
	Yes	77	21	27.3	22	28.6	34	44.1		
Family support during pandemic	No change	163	99	60.7	57	35	7	4.3	57.38	< 0.001**
	Decrease	127	39	30.7	39	30.7	49	38.6		
Follow news about pandemic	Not follow	54	27	50	20	37	7	13		
	Low	73	47	64.4	20	27.4	6	8.2	54.97	< 0.001**
	Moderate	89	49	55.1	31	34.8	9	10.1		
	Continuous	74	15	20.3	25	33.8	34	45.9		

*χ*^2^: Chi square test, *NS* nonsignificant (*P* > 0.05), *Significant (*P* < 0.05), **Highly significant (*P* < 0.001)

Our results showed that not working, low financial status, fear of infection and death by COVID-19, fear of job loss, had job change during pandemic, increase seizures rate during pandemic, increase ER visits, and lack of drug adherence during the pandemic, increase risk of anxiety by 2.06-, 3.16-, 4.39-, 3.02-, 7.07-, 4.36-, 7.06-, 6.58-, and 5.02-fold (odds ratio), respectively (Table 5).

**Table 5:** Binary logistic regression analysis of the predictors of anxiety

Variable	*B*	S.E	Wald	*P*	OR	95% CI	
Age > 40	0.155	0.676	0.053	0.818	0.856	0.228	3.219
Female sex	0.189	0.987	0.918	0.605	1.440	0.836	5.358
Divorced	0.196	0.425	0.357	0.98	1.302	0.365	9.314
Residence	0.126	0.562	0.050	0.823	1.134	0.377	3.411
Live alone	1.202	0432	0.293	0.510	1.724	0.369	6.157
Illiterate	0.718	0.896	0.641	0.423	2.050	0.354	11.878
Not working	2.822	1.106	6.511	0.011*	2.059	1.007	8.520
> 2 children	1.530	0.995	2.368	0.124	0.216	0.031	1.520
Low financial status	1.830	0.786	5.420	0.020*	3.161	2.034	11.749
Positive family history of epilepsy	1.342	0.733	3.348	0.067	1.261	0.620	1.100
Positive family history of psychiatric disorder	1.862	2.163	0.741	0.389	1.155	0.312	10.773
Positive past history of psychiatric disorder	0.212	0.676	0.098	0.754	1.236	0.328	4.652
Age of onset < 17 years	0.250	0.677	0.137	0.712	1.284	0.341	4.843
Duration > 14 years	0.296	1.303	0.052	0.820	1.344	0.104	17.295
Generalized seizers	0.922	0.818	1.270	0.260	2.515	0.506	12.508
Refractory	0.160	0.963	0.027	0.868	1.173	0.178	7.741
> 1 AEDs	1.260	1.923	0.429	0.512	3.525	0.081	15.263
Any time seizures	0.749	2.417	0.096	0.757	2.114	0.019	24.132
Fear of infection by COVID-19	3.381	2.827	1.430	0.023*	4.389	1.115	7.495
Fear of death by COVID-19	3.793	2.999	1.600	0.006*	3.023	1.369	8.040
Close people infection	1.664	0.793	0.910	0.340	1.070	0.324	6.612
Close people death	0.975	0.947	1.060	0.303	1.377	0.059	2.415
Fear of job loss in pandemic	3.048	1.266	5.793	0.002*	7.071	1.761	25.105
Job changes by pandemic	7.186	3.028	3.630	0.018*	4.364	1.491	19.665
Financial changes in pandemic	0.292	0.697	0.176	0.675	1.747	1.190	2.927
Sleep disturbance during pandemic	1.513	0.956	0.322	0.210	1.178	0.972	4.216
Decrease family support during pandemic	0.657	1.006	0.426	0.514	1.929	0.268	13.868
Continuously following news about pandemic	1.056	0.317	0.727	0.325	1.569	0.865	7.517
Increase seizers rate	2.869	0.873	3.791	0.001*	7.057	3.010	25.314
Increase ER visits rate	2.808	0.911	3.498	0.002*	6.579	2.780	18.889
Lack of drug adherence during pandemic	2.723	1.131	3.028	0.003*	5.024	2.603	16.182
Lack of routine follow-up during pandemic	0.820	0.166	0.352	0.297	1.441	0.318	5.610

*SE* standard error, *OR* odds ratio, *CI* confidence interval. *Significant (*P* < 0.05) **highly significant (*P* < 0.001)

## Discussion

To our knowledge, this is the first study that had been conducted to assess anxiety among PWE during COVID-19 pandemic in Egypt.

Our study found that the prevalence of anxiety during the pandemic is 52.4%: 6.9% of the participants had mild anxiety, 26.2% had moderate anxiety, and 19.3% had severe anxiety.

Our finding corresponds to the existing literature. The prevalence of anxiety in PWE varied across studies. In a systematic review of 11 articles that came out between 2011 and 2019 in different countries, Wang and colleagues found that the prevalence of anxiety in PWE ranged from 21.1 to 45% [15].

In a meta-analysis of 27 studies including 3221 PWE, Scott and colleagues, found that the prevalence of anxiety disorder in PWE ranging from 8.1 to 27.3% [16].

The prevalence of anxiety in PWE varied also during the pandemic. In a study that included 141 patients with epilepsy, Salari and colleagues found that 13.5% of patients had experienced a severe level of anxiety during the pandemic [9].

In a cross-sectional study of 151 PWE**,** Abokalawa and colleagues found that 72.2% of PWE reported anxiety during the pandemic [17].

In this study, we found that multiple COVID-19 pandemic-related factors are associated with increased anxiety in PWE. We detected a statistically significant increase in the frequency of anxiety among patients who fear COVID-19 infection, patients who fear death by COVID-19 infection, patients who fear job loss during the pandemic, patients who already had job changes including job loss during the pandemic, patients having sleep disturbances during the pandemic, patients having less family support during the pandemic and patients who follow the pandemic news moderately to continuously.

Our study is consistent with other studies, Van Hees and colleagues found that anxiety in PWE is associated with financial problems during the pandemic [7]. Rudenstine and colleagues found that unemployment, the death of a family member or close friend, a lack of social support, trouble paying monthly rent, and economic uncertainty are all COVID-19-related situations raise the anxiety level in PWE [8]. Salari and colleagues found that the anxiety levels in PWE during the pandemic were higher if one of the relatives had been infected with COVID-19, and in patients with disturbed sleep pattern [9]. Wang and colleagues, found that weaker social support is associated with increased anxiety among PWE [18].

Using binary logistic regression analysis of predictors of anxiety among the studied group, we found that these factors are significantly associated with the increased risk of anxiety in PWE during the pandemic: fear of job loss during the pandemic is (sevenfold increase in the risk of anxiety), increased seizures’ rate during the pandemic (more than sevenfold increase in the risk of anxiety), increased ER visits during the pandemic (6.6-fold increase in the risk of anxiety), lack of drug adherence during the pandemic (fivefold increase in the risk of anxiety), fear of infection by COVID-19 (4.4-fold increase in the risk of anxiety), job changes during the pandemic including job loss (more than fourfold increase in the risk of anxiety), low financial status (3.1 increase in the risk of anxiety), fear of death by COVID-19 infection (threefold increase in the risk of anxiety), being unemployed (twofold increase in the risk of anxiety).

To the best of our knowledge, our study is the first study to assess these variables in PWE as a risk of anxiety during the pandemic.

### Limitations and recommendations

Our study has some limitations, because the exposure and outcome are examined concurrently in a cross-sectional study, there is often no evidence of a causation link between exposure and outcome and longitudinal studies are recommended. Also, we did not categorize participants into epileptic patients with anxiety and epileptic patients without exploring the differences and risk factors regarding sociodemographic and clinical data of epilepsy. Also, we did not stratify age into subgroups and tested against anxiety to reveal the age range most affected with COVID-related anxiety. However, we have many strengths in our study, as our results are useful in focusing on PWE who are already under severe stress that increased more in the pandemic. Our study was performed by direct doctor–patient interview, not online or self-submitted questionnaires, which guarantees correct understanding of the patients to the questions and good interpretation of the results. Our study was performed in an epilepsy clinic not in primary care clinic, allowing us to reach the medical records of the patients, which was very important to confirm the diagnosis, the type of seizures, the duration of illness, the number of anti-seizure medications and the past medical history. PWE should be regularly screened for anxiety especially during unusual circumstances like COVID-19 pandemic. Early detection of anxiety in PWE and early adjustment of its risk factors help in early treatment and better outcomes that will be reflected also on better management of epilepsy and better quality of life for those patients.

## Conclusions

Our study revealed a high prevalence of anxiety in PWE during COVID-19 pandemic. During the pandemic, fear of job loss during the pandemic, increased seizures’ rate during the pandemic, increased ER visits during the pandemic, lack of drug adherence during the pandemic, fear of infection by COVID-19, job changes during the pandemic including job loss (more than fourfold increase in the risk of anxiety), low financial status, fear of death by COVID-19 infection, being unemployed were the most significant predictors for anxiety in PWE. So, these risk factors must be evaluated and adjusted as these will be reflected in the improvement of the anxiety, which in turn will be reflected in the improvement of epilepsy and on the quality of life of PWE.

## Data Availability

Available upon request.

## Abbreviations

PWE: Patients with epilepsy
AEDs: Anti-epileptic drugs
ER: Emergency room
GAD: Generalized anxiety disorder

## References

[1] Fisher RS, Acevedo C, Arzimanoglou A, Bogacz A, Cross JH, Elger CE (2014). ILAE official report: a practical clinical definition of epilepsy. Epilepsia.

[2] Salpekar JA, Mula M (2019). Common psychiatric comorbidities in epilepsy: how big of a problem is it?. Epilepsy Behav.

[3] Kanner AM (2017). Psychiatric comorbidities in new onset epilepsy: should they be always investigated?. Seizure.

[4] Scott AJ, Sharpe L, Thayer Z, Miller LA, Hunt C, MacCann C (2019). Design and validation of two measures to detect anxiety disorders in epilepsy: the Epilepsy Anxiety Survey Instrument and its brief counterpart. Epilepsia.

[5] Horesh D, Brown AD (2020). Traumatic stress in the age of COVID-19: A call to close critical gaps and adapt to new realities. Psychol Trauma.

[6] Xiong J, Lipsitz O, Nasri F, Lui LMW, Gill H, Phan L (2020). Impact of COVID-19 pandemic on mental health in the general population: a systematic review. J Affect Disord.

[7] Van Hees S, Siewe Fodjo JN, Wijtvliet V, Van den Bergh R, de Moura F, Villela E, da Silva CF (2020). Access to healthcare and prevalence of anxiety and depression in persons with epilepsy during the COVID-19 pandemic: a multicountry online survey. Epilepsy Behav.

[8] Rudenstine S, McNeal K, Schulder T, Ettman CK, Hernandez M, Gvozdieva K (2021). Depression and anxiety during the COVID-19 pandemic in an urban, low-income public university sample. J Trauma Stress.

[9] Salari M, Etemadifar M, Gharagozli K, Etemad K, Ashrafi F, Ashourizadeh H (2020). Incidence of anxiety in epilepsy during coronavirus disease (COVID-19) pandemic. Epilepsy Behav.

[10] Liu Z, Yin R, Fan Z, Fan H, Wu H, Shen B (2020). Gender differences in associated and predictive factors of anxiety and depression in people with epilepsy. Front Psychiatry.

[11] Spitzer RL, Kroenke K, Williams JB, Löwe B (2006). A brief measure for assessing generalized anxiety disorder: the GAD-7. Arch Intern Med.

[12] Kroenke K, Spitzer RL, Williams JBW, Löwe B (2010). The Patient Health Questionnaire Somatic, Anxiety, and Depressive Symptom Scales: a systematic review. Gen Hosp Psychiatry.

[13] Terkawi AS, Tsang S, AlKahtani GJ, Al-Mousa SH, Al Musaed S, AlZoraigi US (2017). Development and validation of Arabic version of the Hospital Anxiety and Depression Scale. Saudi J Anaesth.

[14] IBM crop. Released (2017) IBM SPSS statistics for windows, Version 25.0. Armonk, NY: IBM crop.

[15] Wang Z, Luo Z, Li S, Luo Z, Wang Z (2019). Anxiety screening tools in people with epilepsy: a systematic review of validated tools. Epilepsy Behav.

[16] Scott AJ, Sharpe L, Hunt C, Gandy M (2017). Anxiety and depressive disorders in people with epilepsy: a meta-analysis. Epilepsia.

[17] Abokalawa F, Ahmad SF, Al-Hashel J, Hassan AM, Arabi M (2022). The effects of coronavirus disease 2019 (COVID-19) pandemic on people with epilepsy: an online survey-based study. Acta Neurol Belg.

[18] Wang HJ, Tan G, Deng Y, He J, He YJ, Zhou D (2018). Prevalence and risk factors of depression and anxiety among patients with convulsive epilepsy in rural West China. Acta Neurol Scand.

